# Reaching Priority Populations When Scaling Up: A Qualitative Study of Practitioners' Experiences of Implementing Early Childhood Health Interventions in Victoria, Australia

**DOI:** 10.1111/mcn.70046

**Published:** 2025-05-19

**Authors:** Sarah Marshall, Penelope Love, Konsita Kuswara, Karen Lee, Hannah Downes, Rachel Laws

**Affiliations:** ^1^ Prevention Research Collaboration, School of Public Health, Faculty of Medicine and Health, The University of Sydney Sydney New South Wales Australia; ^2^ Charles Perkins Centre, The University of Sydney Sydney New South Wales Australia; ^3^ The Institute of Physical Activity and Nutrition (IPAN), School of Exercise and Nutrition Science, Deakin University Geelong Victoria Australia; ^4^ NHMRC Centre of Research Excellence in Translating Early Prevention of Obesity in Childhood (EPOCH‐Translate CRE) Sydney New South Wales Australia; ^5^ Cohealth Footscray Victoria Australia

**Keywords:** health equity, implementation science, population health, preventive health services, qualitative research, vulnerable populations

## Abstract

Implementing evidence‐based health promotion programmes at scale is important for achieving population‐level health outcomes. However, achieving equitable reach can be challenging. An evidence‐based early‐life nutrition and movement intervention (INFANT) is currently being implemented at scale in Australia. This study explored practitioners' perceptions about reaching priority population groups through INFANT or similar universal preventive health programmes and services implemented at scale. Fifteen semi‐structured online interviews were conducted with purposively selected experienced practitioners. Interviews were transcribed and analysed using reflexive thematic analysis. Two main themes were developed, representing the complexities of reaching priority population groups through universal health programmes and services in early childhood. Theme 1: *“We're not hitting the mark”—Underlying issues influence universal healthcare access*, highlighted (a) the perception that parents felt out of place through a sense of not belonging or being unfamiliar with universal services and (b) that practical constraints and pressing priorities impacted access. Theme 2: *“There are no easy answers”—Varied approaches can enhance engagement, but the path is not straightforward*, encompassed (a) the importance of trust and familiarity with providers, (b) the suitability of programmes and services for target communities, and (c) factors such as practitioner uncertainty regarding approaches that could address the needs of families from priority populations and resourcing that can limit targeted efforts. Our findings highlight the complexities of achieving equitable reach during scale‐up and the varied decision‐making and resourcing for addressing inequity in a local context. While our findings identify local‐level strategies to address equitable reach during scale‐up, we emphasise that striving to achieve health equity should be embedded and prioritised in scale‐up efforts.

## Summary

- Implementing evidence‐based health promotion programmes at scale is important for population‐level health outcomes, but achieving equitable reach can be challenging.
- Our findings reveal a mismatch between universally offered maternal and child health programmes and services and the needs of families from priority populations.
- Practitioners raised some local‐level strategies to engage families from priority populations, though uncertainties exist regarding the best approaches to plan and implement targeted approaches under local resourcing constraints.
- Health equity considerations should be embedded, appropriately resourced, and prioritised in the practice and research of implementing and scaling up preventive health programmes/services. Co‐design with diverse end‐users is essential to achieving this.

## 1 Introduction

Early life is a critical time when child health and development are sensitive to risk and protective factors (Black et al. 2022; Walker et al. 2011). Health behaviours encompassing nutrition and movement behaviours (active play, sedentary behaviour, and sleep) during the early years of life are influential for immediate well‐being and also have impacts extending into adulthood (Birch et al. 2007; Black et al. 2022; Han et al. 2010). It is widely recognised that lower socioeconomic conditions, such as education level, income, and housing, are associated with adverse health and developmental outcomes during these formative early childhood years (Moore et al. 2015). Further to this, access to and utilisation of healthcare services is less likely amongst families from migrant backgrounds, lower socioeconomic status, and rural or remote areas of residency (Dougherty et al. 2020; Fox et al. 2021; Khatri and Assefa 2022; Ou et al. 2011; Taylor and Lamaro Haintz 2018).

Evidence‐based behavioural interventions for supporting families with health behaviours of their children aged 0–5 years are effective in improving health and development outcomes (Brown et al. 2019; Hennessy et al. 2019; Lioret et al. 2023; Redsell et al. 2016). Implementing evidence‐based nutrition and movement health promotion programmes at scale in the critical period of early life is essential for improving population‐level health outcomes and should be prioritised (Vaivada et al. 2017). Achieving equitable reach (i.e., people participating in an intervention irrespective of background and addressing potential inequities in participation) is an important aspect of scaling up a health intervention, but it is also a potential difficulty during scale‐up (Zomahoun et al. 2019).

There is a gap in our understanding of how best to effectively meet the needs of targeted, underserved communities in early‐life health promotion programmes implemented at scale. The disconnection between the programme or services offered and the specific needs of different communities and individuals can result in health inequities. Local adaptations often address this, whereby individual implementation sites decide how best to approach this in their context. It is increasingly acknowledged that this is an essential step in intervention implementation success (Glasgow et al. 2022; Moore et al. 2021). Further to this, while current scalability assessment tools (Ben Charif et al. 2022) and scale‐up process guidance (Milat et al. 2015) encourage consideration of the local adaptations required, they offer limited information about how to incorporate and consider equity at scale.

### 1.1 Context

INFANT (INfant Feeding, Active play, and NuTrition) is an evidence‐based intervention to improve child nutrition and movement behaviours through professional‐led group sessions and a mobile phone app targeting parents and caregivers over the first 18 months of their child's life. In 2008, INFANT was delivered as a randomised controlled trial (Campbell et al. 2008) and showed positive maternal and child outcomes under controlled conditions (Campbell et al. 2013; Lioret et al. 2012). Following a small‐scale translation trial demonstrating proof of concept in 2012 (Laws et al. 2016; Love et al. 2018), from 2020, INFANT is being implemented at scale across Victoria, Australia and evaluated as a hybrid implementation‐effectiveness trial (Laws et al. 2021; Marshall et al. 2022). At the time of collecting data for this study in mid‐2022, 30 (of 79) Victorian Local Government Areas were implementing or planning to implement INFANT in their area, growing to 46 by 2024. Also, notably, at the time of the INFANT scale‐up and this nested study, significant emergency responses were enacted in Victoria, Australia, due to the COVID‐19 pandemic.

### 1.2 The Current Study

This study builds on the planned scale‐up evaluation of INFANT and will expand our understanding of scale‐up approaches for reaching underserved priority populations. This study explored local practitioners' experiences and engagement approaches when delivering INFANT and other universal early‐life health promotion programmes amongst priority population groups. Local practitioners possess extensive expertise and invaluable on‐the‐ground experience, making them essential to local implementation processes. In this study, we refer to priority population groups as those underserved by early‐life health services and at risk of poorer health outcomes, specifically individuals from culturally and linguistically diverse backgrounds (a term commonly used in Australia, defined as people born in non‐English speaking countries, and/or who do not speak English at home; Pham et al. 2021) and those experiencing socioeconomic disadvantage (based on socioeconomic position indicators such as income, education, or area of residency).

## 2 Methods

### 2.1 Study Design

This study involved semi‐structured interviews and reflexive thematic analysis of the data. Aligned with our aim, our analysis focused on practitioners' lived experiences and perspectives. We chose individual semi‐structured interviews to allow for in‐depth exploration of ideas and experiences with each participant (Braun and Clarke 2013). We used reflexive thematic analysis to take a data‐driven approach, recognising the interpretive nature of data analysis and the researchers' active role in the generation of knowledge and understanding (Braun and Clarke 2019, 2021). The Standards for Reporting Qualitative Research (SRQR) were followed to guide the reporting of this study (O'Brien et al. 2014).

### 2.2 Sampling and Recruitment

Eligible participants were early‐years child health service providers implementing or preparing to implement INFANT or those with experience working with priority population groups (families from culturally and linguistically diverse backgrounds, lower income or lower education backgrounds, or living in regional or remote areas). We aimed for representation from metropolitan and regional areas and different service organisations in Victoria, Australia.

We used purposive and snowball sampling techniques to recruit up to 25 participants for interviews. The final number of participants was determined pragmatically based on the practical constraints of the recruitment period and the researchers' judgement of a sample size sufficient to contribute to our understanding of the research question. The INFANT implementation team members identified potential participants across 12 purposively sampled Victorian local government areas (i.e., local administrative divisions) implementing INFANT and considering priority population groups. They then sent the potential participants email invitations on behalf of the lead researcher. The potential participants could forward the invitation to others they thought were appropriate. We also included an advertisement for this study in our newsletter sent to practitioners who completed the INFANT implementation training. Voluntary participation was emphasised. Interested individuals accessed a weblink to review the study information and provide consent (using REDCap data management software). Participants could contact the lead researcher for more information at any time. Recruitment occurred from April to July 2022.

### 2.3 Data Collection

Once consent was provided, a brief online survey collected information about participant characteristics such as job title, location, organisation, years of professional experience, country of birth, and language spoken at home. The lead researcher then scheduled an interview at a time suited to the participant. The interviews aimed to explore practitioners' views on engaging priority population groups through INFANT or other child health services. It was pilot‐tested with one participant and refined. An overview of the questions used in the interview guide is presented in Box 1. Interviews were conducted via Zoom videoconferencing software from May to August 2022. The audio files were recorded with consent.

Box 1 **Summary of the interview guide.**

- Could you start by telling me about your background and your experiences working with families from priority populations?
- Could you tell me about your current or planned practice with INFANT and/or other universal early‐life health services/programmes that reach priority populations?
- Have you already made, or do you plan to make, modifications or adaptations to INFANT and/or other early‐life health services reaching priority populations? Can you elaborate?
- What do you experience as the challenges and enablers for implementing INFANT and/or other universal early‐life health services/programmes amongst priority populations?
- Do you have suggestions about suitable programme resources for priority population groups?
- Do you have any other comments or additional information you would like to share?

### 2.4 Data Management and Analysis

A descriptive analysis of survey data was conducted to summarise participant characteristics. Interview audio files were professionally transcribed, and then S.M. cross‐checked for accuracy and removed identifying information. We used reflexive thematic analysis, following Braun and Clarke's six steps (Braun and Clarke 2006, 2019). NVivo software was used to aid the analysis process and clustering ideas. The analysis process involved first becoming familiar with the data by listening to the audio and taking notes. Then, S.M. and K.K. independently coded two transcripts inductively (chosen for a mix of practitioner location, organisation type, and role), labelling any data useful in addressing the research questions, and then met to discuss and generate ideas. Then, S.M. coded the remaining transcripts. Coding was iterative, with codes being refined and data re‐evaluated throughout the coding process. Once all interviews were coded, all codes and data were revisited, and further coding was applied. Next, we grouped codes for shared meaning across the dataset and developed candidate themes that were reviewed in relation to coded data and the entire dataset, then defined and named. The writing of the report was the final step, though it was interwoven through the analysis process, particularly from the stage of developing candidate themes. Reflective notes were maintained throughout, and regular team meetings were held to generate and challenge ideas.

### 2.5 Researcher Characteristics and Reflexivity

The research team included a range of public health, health promotion, and implementation experts. The lead researcher (S.M.) and two co‐authors (P.L. and R.L.) were directly involved with the INFANT scale‐up research, all holding doctoral qualifications in public health and expertise in qualitative methodologies. Other co‐authors, K.K., K.L., and H.D. were not involved in the INFANT scale‐up. They offered expertise in qualitative research and priority populations (K.K.), scale‐up (K.L.), and local service delivery (H.D.). At the time of this study, S.M. was the project manager for the INFANT implementation‐effectiveness trial and a post‐doctoral fellow. The team members' positions offered both unique advantages and potential challenges. Advantages included a shared connection with participants, their implementation experience, and some prior understanding of engaging priority populations in health programmes. However, disadvantages could include the risk of overlooking aspects due to familiarity and the potential influence of pre‐existing relationships on participant responses. S.M. held a pre‐existing relationship with 11 of the participants through her role with INFANT. To address these challenges, the team employed a strategy of reflexivity, continuously evaluating their perspectives throughout the research process. We acknowledge that our analysis is shaped by our health practice and research training (predominantly positivist science), our belief in evidence‐based interventions and their potential at a larger scale, and our belief in universal health care to improve health for all.

#### 2.5.1 Ethical Statement

This study was approved by the Deakin University Human Ethics Advisory Group, Faculty of Health (reference: HEAG‐H 27_2022).

## 3 Results

### 3.1 Description of Participants

Fifteen practitioners participated (14 interviews were conducted as two practitioners in one local government area opted to participate together). The mean interview duration was 45 min (30–55 min). One practitioner who consented did not complete an interview due to illness. The majority were involved in implementing INFANT (*n* = 11), working at a local council (*n* = 8), and based regionally (*n* = 9). Most practitioners had more than 10 years of experience (*n* = 11). Participants held a range of roles, including Maternal and Child Health Nurse or Coordinator (also known as Child and Family Health Nurse, Community Health Nurse or Public Health Nurse) (*n* = 5), Dietitian (*n* = 3), Parenting or Early Years Practitioner or Coordinator (roles involved in implementing early years programming for supporting the mental, social, emotional, and educational development of children 0–5 years) (*n* = 3) and Health Promotion Officer (*n* = 1). Three practitioners were Programme Officers or Managers from Cultural Support Organisations that are non‐government/third‐sector organisations. Practitioners worked with culturally and linguistically diverse families (*n* = 9) and families experiencing greater needs (*n* = 6). Participant characteristics are presented in Supporting Information S1: File S1.

### 3.2 Thematic Analysis

**Figure 1:**
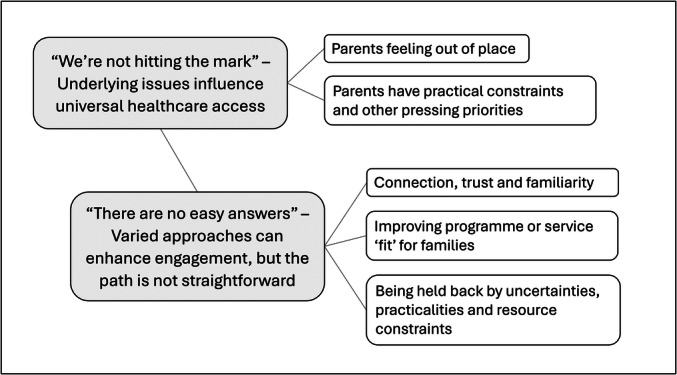
Thematic map.

Two main themes were developed representing the complexities of engaging priority population groups through universal health programmes and services in early childhood: (1) “We're not hitting the mark”—Underlying issues influence universal healthcare access, and (2) “There are no easy answers”—Varied approaches can enhance engagement, but the path is not straightforward. The themes and sub‐themes are described below and illustrated in Figure 1.

#### 3.2.1 Theme 1: “We're not Hitting the Mark”—Underlying Issues Influence Universal Healthcare Access

Theme 1 reflects practitioners' perceptions that universal preventive health programmes and services were not meeting the needs of families from priority populations and that there were underlying root causes of poor engagement. This theme includes subthemes: (a) Parents feeling out of place and (b) Parents have practical constraints and other pressing priorities.

*(a) Parents feeling out of place*

Practitioners expressed perceptions that parents from priority populations felt out of place (i.e., a sense of not belonging or being unfamiliar) within universal services, and this was a barrier to engagement. The sense of feeling out of place included perceived social barriers for parents from priority populations, such as being unfamiliar with staff members, feeling uncomfortable and uncertain, not wanting to be singled out, and feeling unsure if a programme or service was for them. One practitioner expressed difficulties with the group format for accommodating families with diverse resourcing and needs: *“So unfortunately, I would say with some of the more vulnerable families that the nurses would [instead] give the information one‐to‐one because when they are in the group, there are fairly well‐resourced people that would drive the conversation”* (ID12, Early Years Officer, Local Council). Some practitioners indicated there was a power or authority dynamic with practitioners that also contributed to feeling out of place: *“Often, [families from culturally diverse backgrounds] see you in, like, that white coat, you're the expert. So, it's really difficult to break that down”* (ID5, Senior Health Promotion Officer, Community Health Organisation).

*(b) Parents have practical constraints and other pressing priorities*

Practitioners described various practical constraints that parents experienced and their impact on engaging with universal preventive health programmes and services. The constraints identified included costs associated with accessing services, transportation issues, language barriers, work commitments, and scheduling. Additionally, practitioners acknowledged that families may be dealing with more pressing issues, such as severe financial hardship, that take precedence over participating in early‐life preventive health initiatives. For example: *“So, if you've got three kids and a lot going on, going to that nutrition session might not be the top of their needs list at the moment. It might be just getting food to put on the table in the first place”* (ID13, Dietitian, Community Health Organisation).

#### 3.2.2 Theme 2: “There Are no Easy Answers”—Varied Approaches can Enhance Engagement, but the Path Is not Straightforward

Theme 2 reflects practitioners' perceptions that some varied approaches and strategies have proven successful in improving engagement with families from priority populations; however, there is no one answer, and there are factors that can hold practitioners back. This theme includes subthemes: (a) Connection, trust, and familiarity, (b) Improving programme or service ‘fit’ for families, and (c) Being held back by uncertainties, practicalities, and resource constraints.

*(a) Connection, trust, and familiarity*

Practitioners highlighted elements of relationships—connections, trust, and familiarity—that enhanced engagement with families from priority populations. This included linking with other organisations that hold established connections with target communities, facilitators with suitable skills, and using familiar places and less formal approaches to foster ease and an inclusive sense of belonging. Practitioners valued such linkages with appropriate community organisations. These partnerships were valued for offering expertise and trusted, familiar relationships with families from priority populations: *“It's linking into communities where people are already meeting that I think works extremely well”* (ID10, Senior Project Officer, Cultural Support Organisation).

Practitioners also provided examples of programmes or services that successfully engaged priority population groups, often attributing their success to staff members who built trust and familiarity. These staff members possessed essential skills and traits, such as extensive experience, actions that made people feel welcomed and safe, a positive and approachable personality, responsiveness to families, and the ability to skillfully tailor the programme or service to individual needs. *“They have a gentle approach. I think it probably leads to good engagement, and more so for your vulnerable clients, you really need to think about who is working with them”* (ID2, Coordinator Maternal and Child Health, Local Council). Identifying motivated staff was another consideration: *“Some staff embrace different nationalities better than others, some really love engaging with different nationalities […] So, it's just finding the right staff”* (ID8, Maternal and Child Health Coordinator, Local Council). Bicultural workers' roles were specifically discussed as an approach to engaging families from diverse cultural backgrounds. They were perceived as community connectors, facilitating cultural connection, and effective communicators: *“The key to community engagement and participation is the bicultural mentor, who is also of the same cultural background of the cohort of women we want to engage with”* (ID4, Community Programs Manager, Cultural Support Organisation).

Linked to Theme 1, practitioners emphasised the importance of understanding the specific context of the individual to harness trust and apply appropriate approaches, as described by this practitioner: *“We gently support the families, because the first thing they need is that they are feeling okay, their well‐being is okay, and that they have the confidence that what they are doing is a good step […] Starting from where the family is actually at. That was really key”* (ID7, Team Leader Early Years Community Support, Local Council).

*(b) Improving programme or service ‘fit’ for families*

All practitioners discussed some level of adaptation or modification of programmes and services to improve the fit or suitability for families from priority population groups. Tailoring content to individual families was commonly discussed, and this included both planned and in‐session tailoring. Adapting education material, such as accommodating different languages and lower literacy levels, was also discussed. One practitioner described this need to tailor the programme or service to the participants; however, they noted the demand on facilitators to do this: *“You have to individualise to the group. So, it would potentially look different with different families and participants. If you do know the people beforehand, having a bit of a think about what potentially they would require as well, but there's quite a bit on the facilitator to do that”* (ID12, Early Years Officer, Local Council). A practitioner from a cultural organisation emphasised the importance of critically evaluating programmes and seeking guidance when working across different cultural contexts: *“I think you need to work with the ethnic‐specific services, but also you need to look at your program [and] I think you need to be prepared to have it scrutinised, not scrutinised, but assessed culturally”* (ID10, Senior Project Officer, Cultural Support Organisation). Again, specific to families from diverse cultural backgrounds, there were different opinions about whether to establish multicultural groups that are culturally inclusive or single cultural groups that offer in‐depth relevance to the target group. Another commonly suggested modification was adopting a more informal structure like a playgroup (where children play and do activities together while their parents or caregivers supervise and socialise), allowing families to engage in discussions in a relaxed and informal setting. One practitioner described a positive experience taking this approach: *“I had a variety of people in the [playgroup], and the most amazing spontaneous conversations occurred around our fruit snack time. We had so many different conversations around solids and active play. […] if you've got the knowledge and the skills and can weave it into the conversations, the learning often takes place with the parents themselves”* (ID11, Early Parenting Worker, Local Council).

*(c) Being held back by uncertainties, practicalities, and resource constraints*.

There was a sense that practitioners felt strongly that this study was a priority but seemed limited by several factors. A minority of practitioners, with various levels of experience, voiced uncertainty regarding approaches that could address the needs of families from priority populations. For example, one nurse with 20 years of professional practice expressed her hesitations: *“That's a tough one. Look, I'm really not too sure what we could do. […] It's something that we need to think about”* (ID1, Maternal and Child Health Nurse, Local Council). While many did not directly voice this, uncertainties were evident throughout the discussions: *“With INFANT, we'd love to have [culturally diverse families] attending our typical INFANT sessions, but the logistics of having three languages and three interpreters in one room, obviously, is a really a big challenge*” (ID13, Dietitian, Community Health Organisation). A few practitioners also referred to the practicalities, noting that the engagement and support process takes more time and effort, and the planning and practical considerations are greater. While in agreement, one experienced practitioner from a cultural support organisation offered a divergent comment about this: *“Of course, some services are going to go, “It's too hard.” But I think really, in a multicultural society, that's just not good enough”* (ID14, Senior Project Officer, Cultural Support Organisation). Funding and staffing constraints were significant limitations, particularly funding for staff time,staff turnover and the necessity to find individuals with unique skill sets to fill specific roles. Several practitioners also highlighted the benefit of management support and alignment with organisational priorities; however, despite having this support, practitioners reiterated that resourcing ultimately dictated their ability to carry out targeted services effectively.

## 4 Discussion

Our results highlight the need for considered planning and delivery efforts to engage families from priority population groups who are typically underserved by early‐life preventive health programmes or services. The mismatch of existing universal preventive services and the needs of families, and also the varied approaches and challenges of enhancing engagement were salient themes in our analysis. Our findings emphasise the complexities of achieving equitable reach during scale‐up of an early‐years intervention and the varied decision‐making and resourcing for addressing equitable access in local contexts.

### 4.1 Acknowledging Parent‐Level Constraints

Our study highlights that practitioners perceived that universal preventive health services and programmes for early childhood are not adequately meeting the needs of families from priority populations. Health service utilisation studies in Australia support this finding, indicating that families experiencing the greatest healthcare needs are less likely to access services (Fox et al. 2021; Khatri and Assefa 2022; Ou et al. 2011; Taylor and Lamaro Haintz 2018). There are both demand (population or person level) and supply (health service or system level) access issues that underpin this inequitable health service utilisation (Levesque et al. 2013). Within our findings, practitioners perceived parent‐level factors as a key influencer to healthcare access. The literature supports the view that parents feel out of place through a lack of belonging or unfamiliarity with universal services, where factors such as cultural congruency, trusting relationships, and a sense of service suitability are important factors for accessing services (Bonakdar Tehrani et al. 2022; Bonevski et al. 2014; Jones et al. 2017; Marshall et al. 2021). Practical constraints and other pressing priorities for parents are also established in the literature, such as financial burden and transport issues (Bonakdar Tehrani et al. 2022; Bonevski et al. 2014; Jones et al. 2017; Marshall et al. 2021). To gain a deeper understanding of this, future qualitative studies with families from diverse populations should explore their experiences with universal services. Open access publishing facilitated by The University of Sydney, as part of the Wiley ‐ The University of Sydney agreement via the Council of Australian University Librarians.

### 4.2 Building Connection, Trust, and Familiarity

The need for connection, trust, and familiarity underscores the importance of relationships for promoting successful engagement with priority population groups. There are elements here that align with the principles of community engagement (Cyril et al. 2015; WHO World Health Organization 2020) and community‐based participatory research (Berge et al. 2009), such as trust‐building and community partnerships. Community partnerships, particularly organisations with existing connections with target communities, have been identified as a key element in engaging priority population groups in health services (Boothroyd et al. 2017; Cyril et al. 2015). Close partnerships between health promotion organisations and community, cultural, and social services are essential for building programme social capital and potentially facilitating integration with universal services. One example of improving developmental surveillance in young children from priority populations in New South Wales, Australia, illustrates how strategic partnerships across local health services and non‐government organisations, built on trust and shared goals, can lead to successful integrated models of care and improved healthcare access (Edwards et al. 2020).

Practitioners emphasised the importance of individual practitioners with appropriate skills, experience, and trust with community members. The use of peers or members of the target group, such as bi‐cultural workers, for intervention delivery has been shown to improve engagement and effectiveness, fostering cultural safety and belonging (Bonevski et al. 2014; Cyril et al. 2015; O'Mara‐Eves et al. 2015). A successful example of this is the implementation of the Cross‐Cultural Workers in Maternity and Child and Family Health Services in Sydney, Australia, which demonstrated positive outcomes for practitioners and families, and is now integrated into local services (Rogers et al. 2023).

### 4.3 Improving the Suitability of Services and Programmes

This study identified some key strategies discussed by experienced local practitioners to improve the fit of early childhood programmes and services for priority population groups. Practitioners commonly suggested tailoring programme content to improve appropriateness amongst target communities, which is a common strategy for programme adaptations (Escoffery et al. 2018; Marshall et al. 2021). Changes to the programme format and setting were also common strategies, such as meeting where target communities already gather or finding new avenues to integrate with other services. For example, supported playgroups may serve as an opportunistic entry point to reach priority groups, as highlighted by a recent study examining health behaviour conversations at playgroups (Middleton et al. 2024).

Interventions often require considerable adaptations for scale‐up, and the need for local flexibility and contextual implementation is increasingly recognised (Barrera et al. 2017; Gillespie et al. 2015; Glasgow et al. 2022; Moore et al. 2021). This has been highlighted in the scale‐up of other nutrition programmes in high‐income and low‐middle‐income countries. For example, a systematic review by Sutherland et al. (2022) found that of 10 early‐life nutrition interventions implemented and evaluated at scale, all underwent adaptations for scaling, yet only two made cultural adaptations for improving cultural fit. A recent 2024 study found that inequalities exist in large‐scale breastfeeding programmes in Bangladesh, Burkina Faso, and Vietnam and highlighted the high priority for introducing equity‐specific actions in scale‐up efforts (Sanghvi et al. 2024). Studies investigating international scaled‐up child development and nutrition programmes found that consideration of implementation context and appropriate and iterative adaptations (codesigned or informed by community needs) were required for optimising scale‐up generally and specifically to reach priority population groups (Buccini et al. 2023; Gillespie et al. 2015; Hernández‐Cordero et al. 2022).

Considering intervention and implementation strategy adaptations before and during implementation is a valuable approach to improving the suitability and reach of health services and interventions (Miller et al. 2021; Wiltsey Stirman et al. 2019). Key to adaptation and learning from local practitioners is the careful planning, measurement and documentation of adaptations and implementation processes to understand what and how such changes influence programme effectiveness outcomes and implementation outcomes (Alvidrez et al. 2019; Durlak and DuPre 2008). Further to this, appropriate adaptations can also lead to better sustainability of interventions over the longer term (Shelton et al. 2018).

### 4.4 Considering Uncertainties and Resourcing for Local Implementation

As commonly referred to in the literature, there is no ‘one‐size‐fits‐all approach' for meeting the needs of diverse priority population groups (Shelton and Brownson 2024), and the nature of a contextually specific approach is complex (Hawe et al. 2009). The uncertainty in how to achieve equitable service delivery expressed by practitioners in our study could reflect the scale of the challenge and the demands on practitioners to consider and execute local‐level programme implementation. This also relates to the constraints of limited time and resources that practitioners described. Because this study was conducted during the COVID‐19 pandemic, and healthcare systems were under immense strain, it is possible that the organisational resourcing constraints expressed by practitioners may have been accentuated. Irrespective of this, the reliance on local‐level responses to achieve equitable service delivery and meet the needs of all families must be addressed with strengthened resources at a system level. A 2023 study highlighted a significant policy‐implementation gap in culturally responsive care for migrant maternal health, also identifying service providers' experience of tensions to achieve this and the need for structural change (Olcoń et al. 2023).

Organisational‐level factors identified in our study, primarily organisational resourcing and leadership support, were aligned with those identified for the sustained delivery of INFANT across mainstream services (Love et al. 2022). Prioritising and mobilising resourcing could be a cost‐effective strategy (Killedar et al. 2023) and is an essential enabling factor for equitable health coverage (Gwatkin et al. 2004; WHO World Health Organization 2020) and intervention scale‐up (Gillespie et al. 2015; Milat et al. 2015). The role of organisational management and decision‐makers in supporting intervention and service delivery that meets the needs of priority populations and achieving health equity is well documented; for example, establishing an organisational culture that values equity, generating buy‐in, identifying and mobilising capacity, and securing on‐going investment (Aarons et al. 2016; Doherty et al. 2022; Vidgen et al. 2018; Whelan et al. 2018).

In addition to local organisational‐level change, there must also be appropriate system and policy solutions to support equitable health services in response to context, for example universally subsidised access to health care, integrating pathways into universal care systems and investing in appropriate resourcing (Chin et al. 2012; Doherty et al. 2022; Fisher et al. 2022). Proportionate universalism is an approach that addresses the social gradient in health, defined by Marmot et al. 2010 (Marmot et al. 2010) as universal interventions and health actions that are responsive in scale and intensity to the level of disadvantage. Successful examples of proportionate universalism in early childhood health services in Australia include the Victorian Enhanced Maternal Child Health service (Victorian Department of Health 2019) and the Maternal Early Childhood Sustained Home‐Visiting programme in New South Wales (Kemp et al. 2017; Kemp et al. 2011). Such solutions require political commitment and all government actions to achieve healthcare equity.

### 4.5 Implications for Practice and Future Research

There is increasing dialogue regarding equity considerations in implementation and scale‐up research (Baumann and Cabassa 2020; Eslava‐Schmalbach et al. 2019; Fort et al. 2023; Gustafson et al. 2023; Loper et al. 2021; McLoughlin and Martinez 2022; Woodward et al. 2021), with existing scale‐up tools and frameworks (Ben Charif et al. 2022; Milat et al. 2015) offering limited direction for reaching priority populations. Our findings can be translated into recommendations for local‐level organisations and practitioners to enhance the equitable reach of universal early years health programmes and services, refer to Box 2. Additionally, we reiterate the need for capacity building of the primary healthcare workforce to promote skills for equitable implementation of programmes/services (Baumann et al. 2023).

Box 2 **Recommendations for local‐level health service organisations and practitioners.**

1. Involve the target community in inclusive and meaningful ways: identify preferences and specific needs, codesign, plan, and deliver with the target community members.
2. Identify and establish relevant partnerships (e.g., community organisations, cultural groups, and community leaders) that can provide insights about community needs, demonstrate established trust with target groups, and facilitate programme delivery.
3. Adapt the programme delivery format, schedules, locations, promotional materials, and recruitment pathways to meet the preferences and needs of the target communities.
4. Adapt programme content and materials to meet the preferences and needs of target communities.
5. Establish trust and rapport with community members. Be empathetic, responsive to needs, respectful, and appreciative of differences.
6. Seek management support and organisational endorsement, including integration into organisational plans and roles.
7. Advocate for adequate organisational resourcing, including funding, staff, and time.
8. Build workforce capacity skills related to equitable implementation, for example, equity‐focused implementation strategies.

### 4.6 Strengths and Limitations

A strength of this study was the use of qualitative methodology, which enabled a deeper exploration of the complexities of reaching priority populations as experienced by practitioners (Hamilton and Finley 2019). Our sampling strategy helped us identify experienced practitioners well‐suited for addressing the research questions. Our reflexivity and our team involvement with analysis strengthened our design by considering biases, building on ideas, and challenging notions (Malterud 2001). A limitation of this study was that due to the nature of snowball recruitment, we interviewed practitioners who focused on culturally and linguistically diverse populations moreso than populations experiencing socioeconomic disadvantage. Another limitation is that at recruitment and data collection, practitioners knew that researchers S.M., P.L., and R.L. were directly involved with the INFANT scale‐up research. While anonymity was assured, this could have potentially affected the information practitioners felt comfortable sharing during the interviews.

## 5 Conclusion

Achieving equitable reach when implementing universal programmes and services at scale is complex. Practitioners in this study identified a mismatch between the services offered and the diverse needs of families from priority population groups. While successful local‐level strategies were identified, such as strong relationships with providers and the suitability of a programme or service, the specific strategies varied and depended on local experiences, context, and resourcing. Future preventive health programme implementation and research should prioritise health equity considerations for reaching priority population groups.

## Author Contributions

S.M., P.L., and R.L. conceptualised this study and designed the methodology. S.M. undertook data collection, led the data analysis, and drafted the manuscript. R.L. and P.L. provided supervision. All authors contributed to data analysis and interpretation. All authors contributed to writing, reviewing, and editing the manuscript. All authors approved the final version.

## Conflicts of Interest

The authors declare no conflicts of interest.

## Supporting information

Supporting file.

## Data Availability

The data that support the findings of this study are available from the corresponding author upon reasonable request.
