# Supplementary material for: Reaching Priority Populations When Scaling Up: A Qualitative Study of Practitioners' Experiences of Implementing Early Childhood Health Interventions in Victoria, Australia
Source: Matern Child Nutr. 2025 May 19;21(4):e70046. doi: 10.1111/mcn.70046 (PMC12454184; doi:10.1111/mcn.70046)
Supplement: Supplementary file 1 — Supporting file. [file MCN-21-e70046-s001.docx]

# Supplementary File

**Table 1.** Sociodemographic characteristics of practitioners (n=15).

| **Sociodemographic characteristics** | **n** |
| --- | --- |
| **Role** |  |
| Maternal and Child Health Nurse and/or Coordinator | 5 |
| Dietitian | 3 |
| Parenting or Early Years Practitioner and/or Coordinator | 3 |
| Program Manager or officer | 3 |
| Health Promotion Officer | 1 |
| **Organisation type** |  |
| Local Council | 8 |
| Community Health Organisation | 4 |
| Cultural Support Organisation | 3 |
| **Working within Victorian Local Government Area(s)** |  |
| Regional | 9 |
| Metropolitan | 6 |
| **Years of experience in professional practice** |  |
| ≤ 10 years | 4 |
| > 10 years | 11 |
| **Country of Birth** |  |
| Australia | 12 |
| Other | 3 |
| **Language mostly spoken at home** |  |
| English | 14 |
| Other | 1 |
| **Involved with implementing INFANT?** |  |
| Yes | 11 |
| No | 4 |
